# Single-stage full-depth scalp reconstruction with Matriderm®: a clinical case report and a brief literature review

**DOI:** 10.1080/23320885.2024.2342329

**Published:** 2024-05-06

**Authors:** Christos N. Noulas, Markos A. Markou, George I. Voulgaris, Eleni I. Effraimidou, Nikolaos A. Papadopulos

**Affiliations:** a First General Surgery Department, University Hospital of Alexandroupolis, Democritus University of Thrace, Alexandroupolis, Greece; b Department of Plastic Surgery and Burns, University Hospital of Alexandroupolis, Democritus University of Thrace, Alexandroupolis, Greece

**Keywords:** Vertex deficit, dermal regeneration template, scalp reconstruction, Matriderm®, skin cancer

## Abstract

Reconstructing scalp defects after basal cell carcinoma removal in elderly patients is challenging. This case report emphasizes Matriderm® as a successful alternative, addressing limitations of traditional methods. The application of Matriderm® in resource-limited scenarios adds insights to surgical literature, and its’ usage addresses challenges in patients, contributing to surgical knowledge.

## Introduction

The incidence of basal cell carcinoma (BCC), a subtype of non-melanoma skin cancer, is high worldwide. Incidents of BCC are commonly light-skinned individuals, with increased age and prolonged exposure to ultraviolet radiation. BCC is not considered an aggressive malignancy, since it counts fewer than 0.1% to 2% of all patient cancer fatalities [1,2].

BCC typically occurs in sun-exposed skin lesions and is usually found in the head and neck. It grows in the basal cell layer of the epidermis, where UV rays damage DNA, which is where thymine dimers are formed, and mutagenesis occurs. Various therapeutic alternatives are available for patients who are not candidates for surgery, including topical treatments with 5-fluorouracil [1,3] or systemic drugs such as vismodegib [2]. Surgical excision is considered the gold standard method since it is the most effective and least expensive treatment for BCC, however it frequently results in scars and soft tissue defects that can be aesthetically disfigured. Due to the risk of tumor recurrence and the requirement for adjuvant radiation therapy, reconstruction after full-depth defects can be demanding and involves skin grafting, local flaps, tissue expansion, and even free flaps [4].

Dermal regeneration templates (DRTs) have become widely used in clinical settings during the past years for the treatment of extensive wounds, burns, and multilayer reconstructions. Matriderm® is a dermal substitute which is a highly porous membrane made of collage and elastin, derived from the bovine dermis and bovine nuchal ligament by hydrolysis respectively [5]. Matriderm® provides a scaffold which helps fibroblasts promoting neovascularization and cell migration. Following implantation of a Dermal Regeneration Template, skin regeneration goes through four distinct histological phases: scaffold imbibition, fibroblast migration, neovascularization, and remodeling with maturation. After fibroblasts generate collagen, Matriderm® is absorbed over a period of six weeks, resulting in the restoration of the skin and modulation of scar formation, reducing the risk of infections and hematomas [6]. The purpose of this case report was to describe a challenging situation involving a full-depth scalp skin defect that was reconstructed using Matriderm® dermal substitute and to provide a brief literature review on full-depth scalp reconstruction using only a DRT. This case report has been reported in line with the SCARE Criteria [7].

## Case report

**Figure 1.**
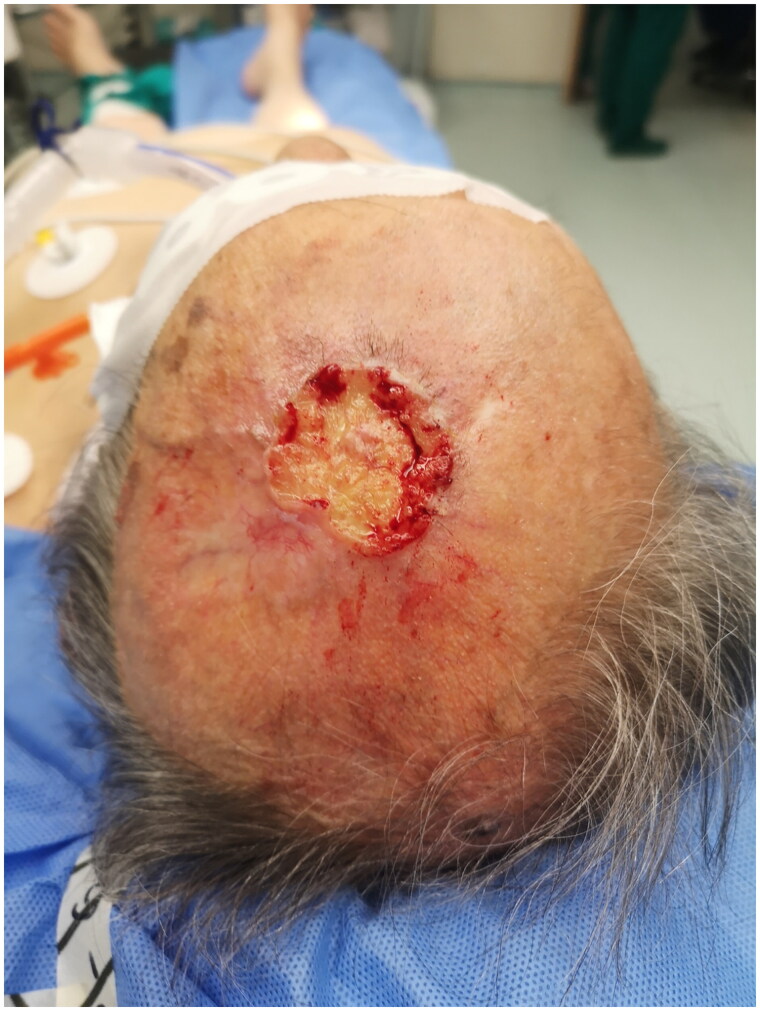
BCC in Vertex of the head.

**Figure 2.**
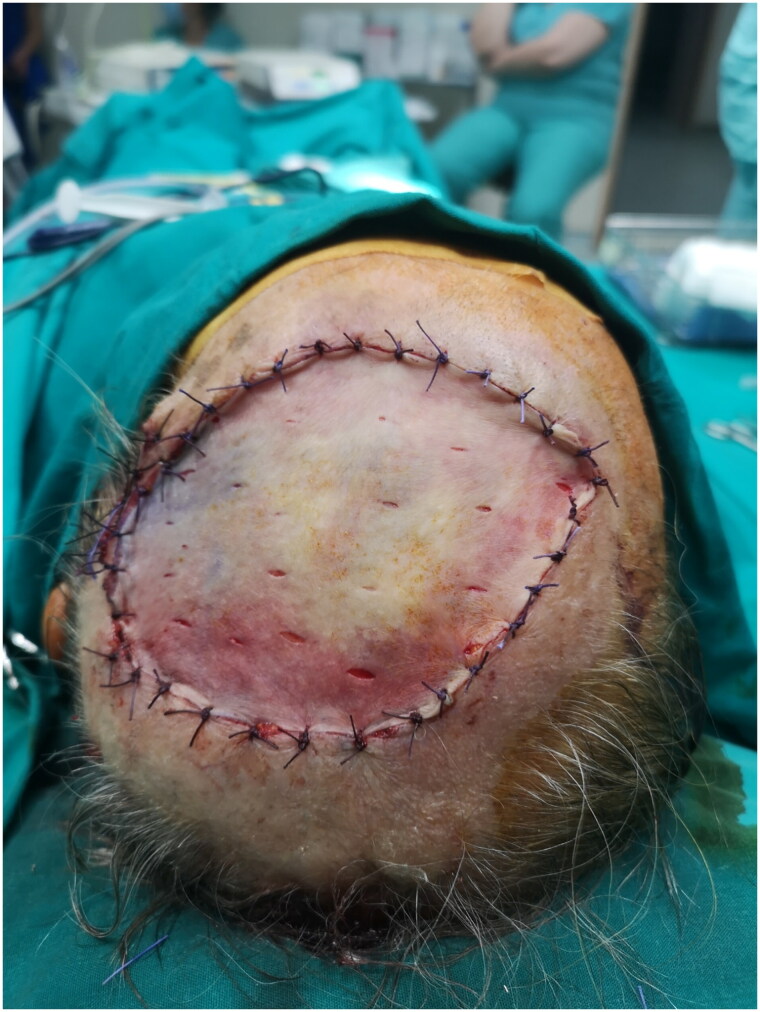
Split-thickness skin graft.

An 83-year male Greek retiree with overall poor health status was referred to our plastic surgery outpatient clinic for a BCC in Vertex of the head. Patient had undergone an incisional biopsy elsewhere (Figure 1). An excisional biopsy was then performed, which confirmed the previously reported BCC of surface spreading type. Clear and adequate margins were achieved. The defect was covered with a split-thickness skin graft, which healed decently (Figure 2).

**Figure 3.**
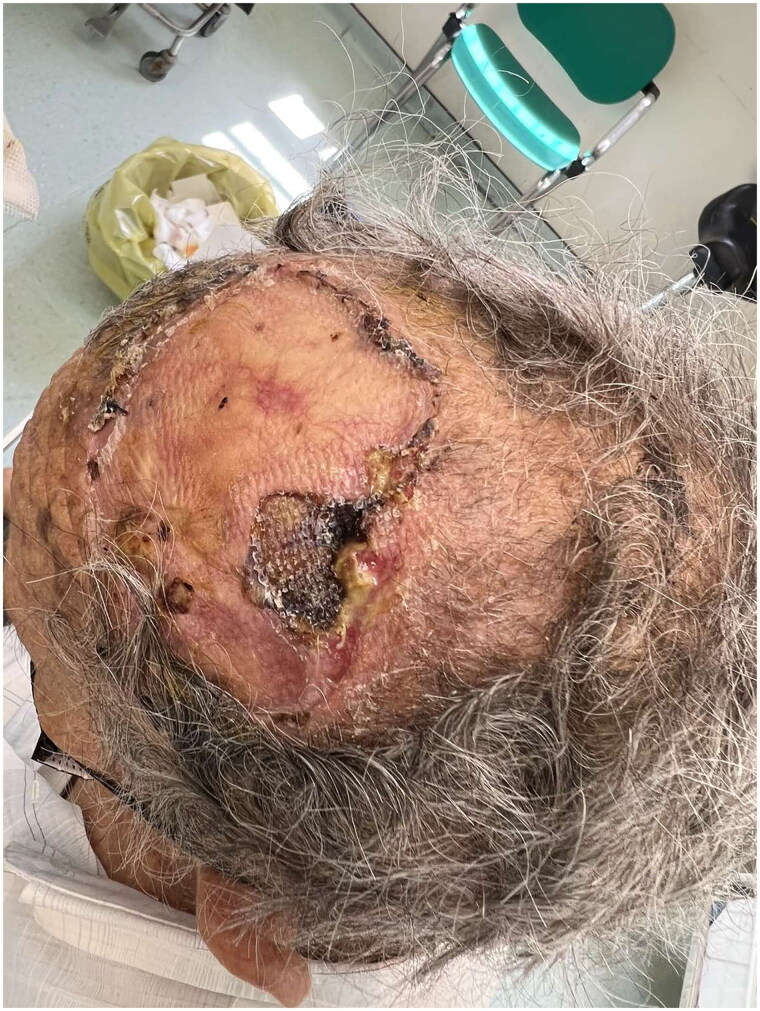
Recurrence of BCC.

**Figure 4.**
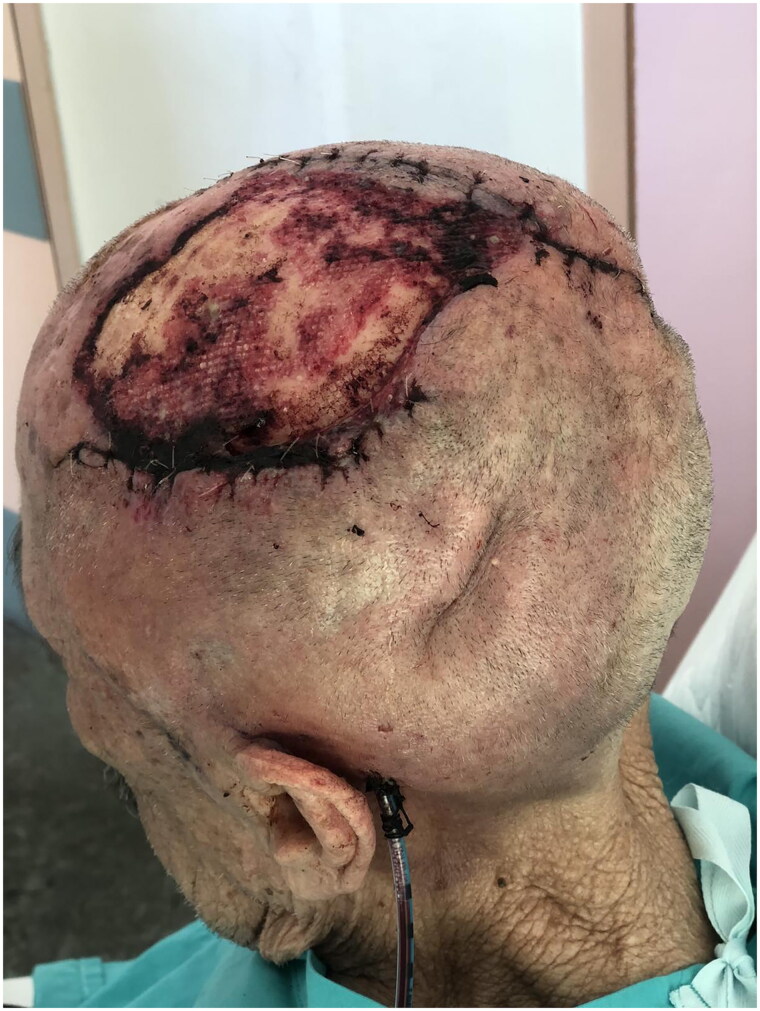
Tissue expanders and Matriderm® applied.

Four months later, the patient was referred again to the outpatient clinic worried about the appearance of the graft (Figure 3). BCC recurrence was clinically suspected, and a new intervention was planned with wider excision, in which the periosteum was also removed. The full thickness deficit of the vertex (12 × 12.5 cm) was then decided to be covered using the tissue expansion method. Two tissue expanders were used to create excess hairy skin. Matriderm® was also used as a temporary cover for the scull (Figure 4).

**Figure 5.**
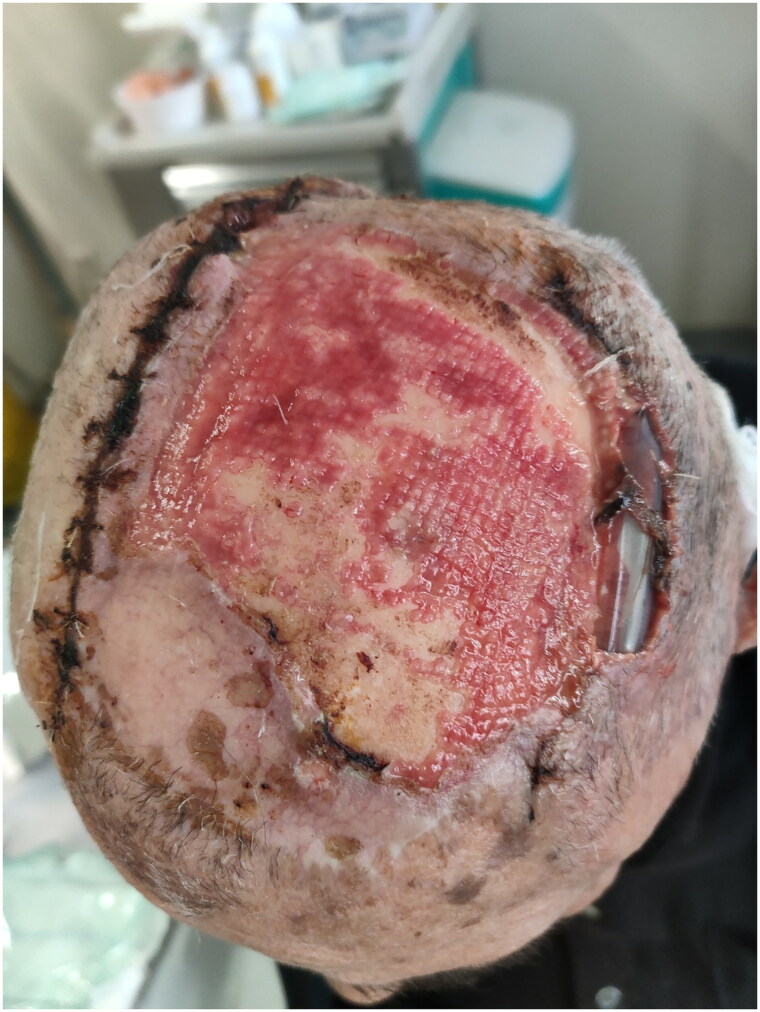
Rejection of expanders.

The expanders were infected and rejected (Figure 5). A culture revealed Pseudomonas aeruginosa, and proper antimicrobial treatment was initiated. The patient was directed toward the operating theater, where the tissue expanders were explanted.

**Figure 6.**
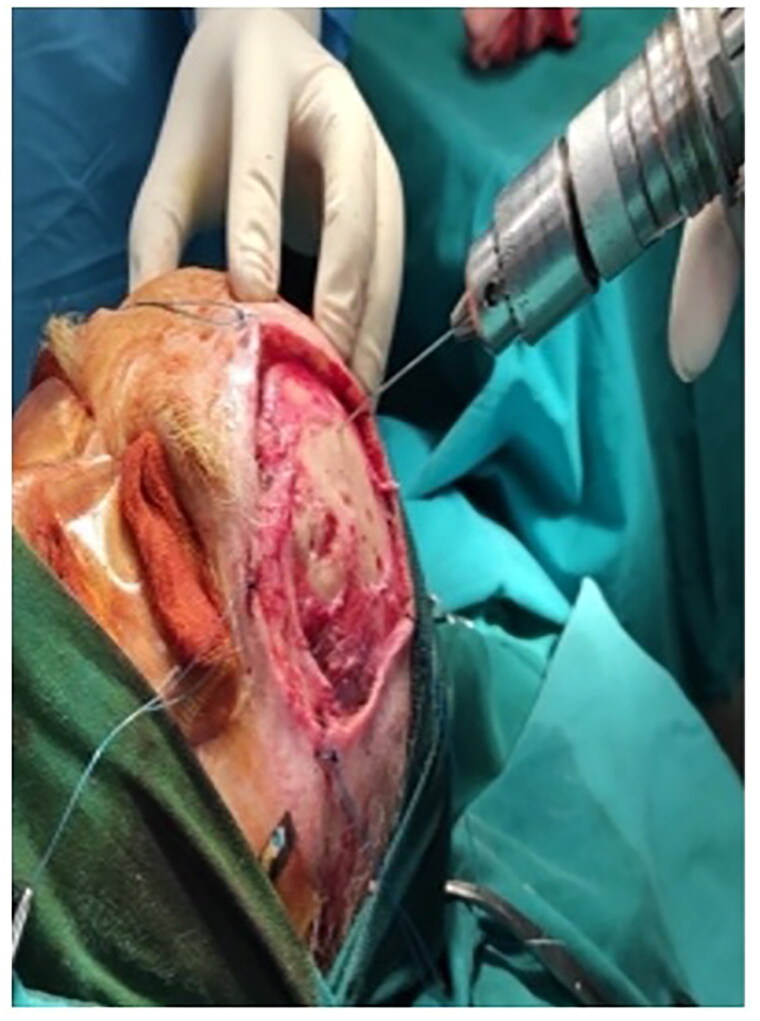
Deploy burring.

**Figure 7.**
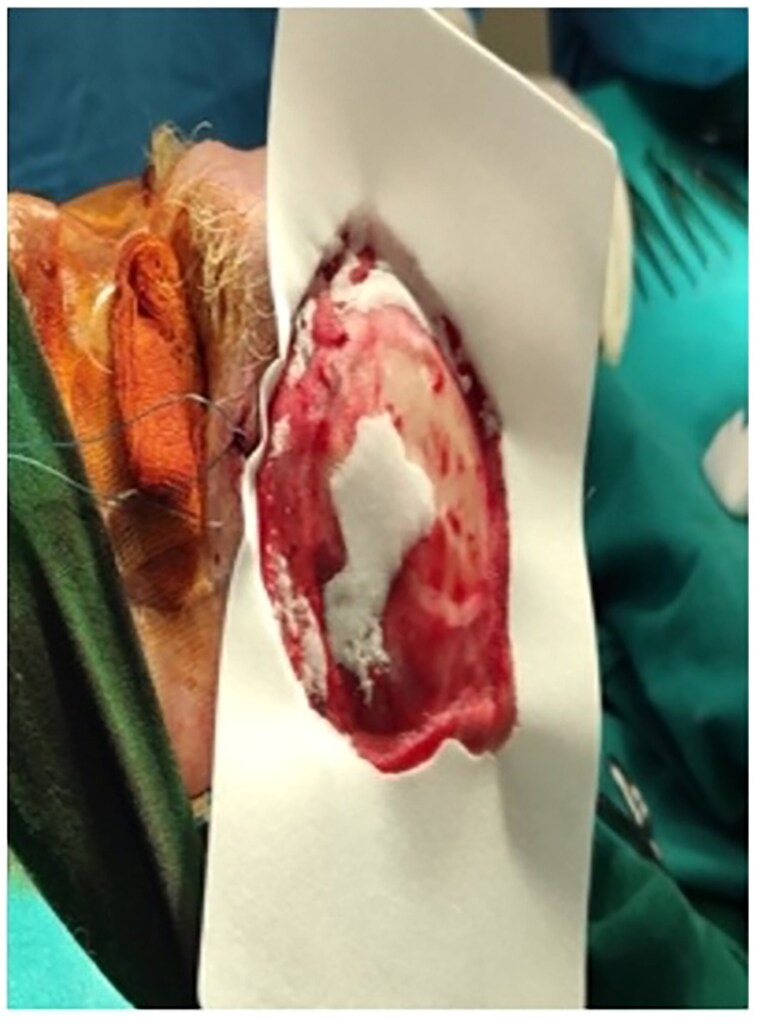
Application of Matriderm®.

The outer compact bone was burred through to the deploy until bleeding for vascularization of DRT (Figure 6). Matriderm® was installed (Figure 7) as a salvage solution since the free flap technique was not an option due to patient’s poor health status. Negative pressure wound therapy (NPWT) in combination with a DRT is a safe and effective option that accelerates and promotes healing [8]. Due to patient’s poor health status, lack of discipline and supportive environment a NPWT system option seemed impractical. Consequently, it was advisable to the patient to continue the post-operative care in an outpatient setting for optimal recovery.

**Figure 8.**
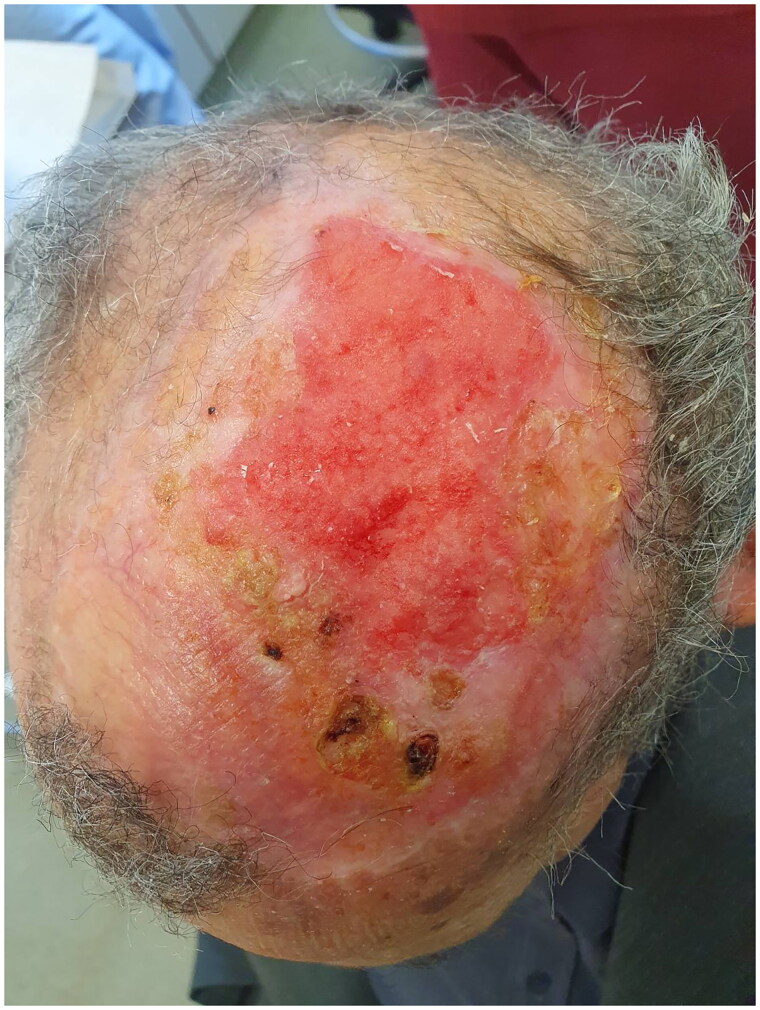
Four months Matriderm® placement.

Four months later, since the patient was subjected to everyday wound care at the outpatient clinic, the deficit seemed to be totally epithelized, innervated, and aesthetically acceptable, considering the age of the patient (Figure 8).

## Literature review

**Figure 9.**
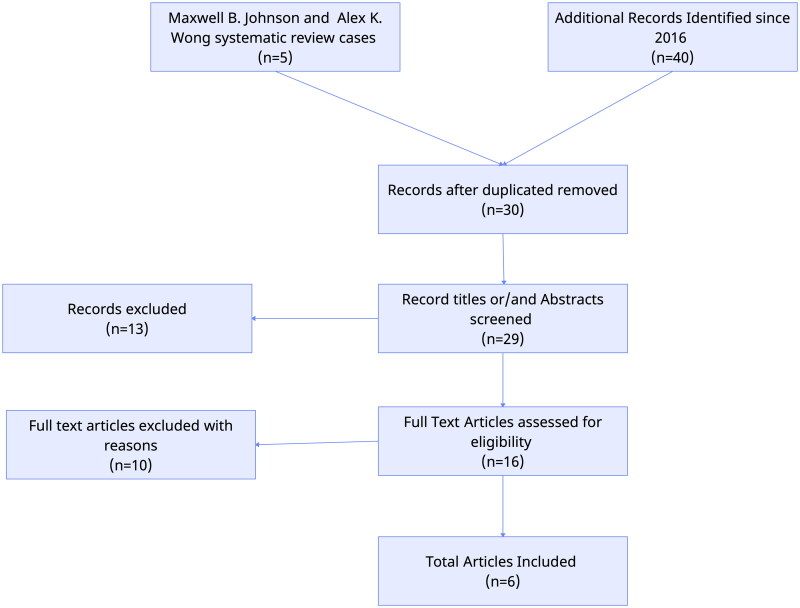
Flowchart of literature’s systematic review search results.

Johnson and Wong 2016 [9] reviewed the literature and found only three articles describing the reconstruction of full-thickness deficits with DRT alone. In our literature review we aimed to identify articles and case reports about one-stage reconstruction of full-depth scalp deficits using only DRTs. We searched on Pubmed using the terms: (((skin substitute) OR (Matriderm) OR (dermal regeneration template)) AND (full thickness scalp)) AND ((one stage) OR (single stage))), ((skin substitute) AND (full thickness scalp)) AND (single stage), ((Matriderm) AND (full thickness scalp)) AND (single stage), (Matriderm) AND (full thickness scalp), (skin substitute) AND (full thickness scalp) as long as these articles were published after June 2016. Cases in which skin grafts or flaps were used, cost analysis papers, *in vitro* studies, and deficits not including the periosteum were excluded. The initial search had no publication type or language limits, and the search engine showed results from 2016 to present. Duplicates were excluded from the study. The numbers of cases reviewed in this study are presented in Table 1. After an initial search of PubMed, 24 articles were retrieved. Thirteen articles were considered ineligible after screening the titles and abstracts. For the remaining 11 publications, the full texts were reviewed for eligibility, and finally, three articles were included in this search. The number of studies reviewed is shown in Figure 9.

**Table 1.** Publications mentioning reconstruction of full-depth scalp deficits using only DRTs in the literature.

Authors	DRT used	Size of deficit	Cause of deficit	Follow up Duration	Number of cases	Sex (M/F)	Comorbidities	Age
Vithlani et al. [11]	Integra®	96.7 cm^2^	SCC & Melanoma	14 months	1	M	N/M	87
Romano et al. [12]	Integra®	–	Spinocellular carcinoma	5 - 7 months	3	N/M	Cardiolovascular problems	76-96
Burd et al. [13]	Integra®	2.85 cm^2^	SCC	23 months	1	M	N/M	86
Singer et al. [14]	Integra®	50 cm^2^	Trauma	N/M	1	M	–	7
Gironi et al. [15]	N/M	25.1 cm^2^	BCC	2 months	1	F	General status	75
Xianghong Lou et al. [10]	Pelnac®	12.5 cm^2^	Trauma	5 months	1	F	–	4

Ultimately, six publications were included in this review, consisting of eight patient cases. Integra® (Integra LifeSciences, Plainsboro, New Jersey) was used in six cases, whereas Lou et al. reported the application of Pelnac® (Gunze Corp., Osaka, Japan) in one patient [10]. The mean size of scalp defects (including the periosteum) was 37.43 cm^2^. Six defects were created due to excision of the malignancies and two due to trauma. The mean follow-up duration was approximately 9 months.

## Discussion

Scalp reconstruction after BCC removal can be demanding, particularly in elderly individuals. When scalp defects occupy a sizable area of the skull, a region of high tension, surgical procedures such as skin grafting, local flaps or tissue expansion may be insufficient.

In this case, all three reconstruction options were applied. First, the skin grafting reconstruction after four months relapsed and BCC reoccurred. Following the second BCC removal of a larger area resulting in a full-depth scalp defect, free flap was not an option because of the patient’s health issues. Therefore, the tissue expansion technique was applied, and the patient developed an infection several days later. Two months after Matriderm® was deployed, the patient’s condition improved, the skin substitute was unified into the wound area, and finally, the wound underwent neovascularization.

A review of the literature revealed that there are not many cases worldwide discussing the reconstruction of a scalp full-depth defect, including the periosteum, with only a DRT, let al.one Matriderm®, so safe and general conclusions cannot be drawn. However, we could state that DRTs may be used to reconstruct small-to-fairly large scalp defects. Skin substitutes, such as Matriderm®, have been used in oncologic and trauma patients of the elderly or young. The follow-up time was not found to be coherent between the cases. DRTs may be used when patients suffer from serious comorbidities, and their general status cannot justify major surgeries, such as free flaps. In periods when an operating time is hardly available, DRTs might help reduce the operating time. Hospitalization can also be reduced resulting in social and economic benefits [16,17].

## Consent

Written informed consent have been obtained
